# The effects of statins on the mevalonic acid pathway in recombinant yeast strains expressing human HMG-CoA reductase

**DOI:** 10.1186/1472-6750-13-68

**Published:** 2013-08-30

**Authors:** Agata Maciejak, Agata Leszczynska, Ilona Warchol, Monika Gora, Joanna Kaminska, Danuta Plochocka, Monika Wysocka-Kapcinska, Dorota Tulacz, Joanna Siedlecka, Ewa Swiezewska, Maciej Sojka, Witold Danikiewicz, Norbert Odolczyk, Anna Szkopinska, Grazyna Sygitowicz, Beata Burzynska

**Affiliations:** 1 Institute of Biochemistry and Biophysics, Polish Academy of Sciences, Pawinskiego 5A, 02-106 Warsaw, Poland; 2 Institute of Organic Chemistry, Polish Academy of Sciences, Warsaw, Poland; 3 Department of Biochemistry and Clinical Chemistry, Medical University of Warsaw, Warsaw, Poland

**Keywords:** HMG-CoA reductase, Statins, Yeast expression system, Heterologous proteins, Mevalonate pathway

## Abstract

**Background:**

The yeast *Saccharomyces cerevisiae* can be a useful model for studying cellular mechanisms related to sterol synthesis in humans due to the high similarity of the mevalonate pathway between these organisms. This metabolic pathway plays a key role in multiple cellular processes by synthesizing sterol and nonsterol isoprenoids. Statins are well-known inhibitors of 3-hydroxy-3-methylglutaryl-CoA reductase (HMGR), the key enzyme of the cholesterol synthesis pathway. However, the effects of statins extend beyond their cholesterol-lowering action, since inhibition of HMGR decreases the synthesis of all products downstream in the mevalonate pathway. Using transgenic yeast expressing human HMGR or either yeast HMGR isoenzyme we studied the effects of simvastatin, atorvastatin, fluvastatin and rosuvastatin on the cell metabolism.

**Results:**

Statins decreased sterol pools, prominently reducing sterol precursors content while only moderately lowering ergosterol level. Expression of genes encoding enzymes involved in sterol biosynthesis was induced, while genes from nonsterol isoprenoid pathways, such as coenzyme Q and dolichol biosynthesis or protein prenylation, were diversely affected by statin treatment. Statins increased the level of human HMGR protein substantially and only slightly affected the levels of Rer2 and Coq3 proteins involved in non-sterol isoprenoid biosynthesis.

**Conclusion:**

Statins influence the sterol pool, gene expression and protein levels of enzymes from the sterol and nonsterol isoprenoid biosynthesis branches and this effect depends on the type of statin administered. Our model system is a cheap and convenient tool for characterizing individual statins or screening for novel ones, and could also be helpful in individualized selection of the most efficient HMGR inhibitors leading to the best response and minimizing serious side effects.

## Background

**Figure 1:**
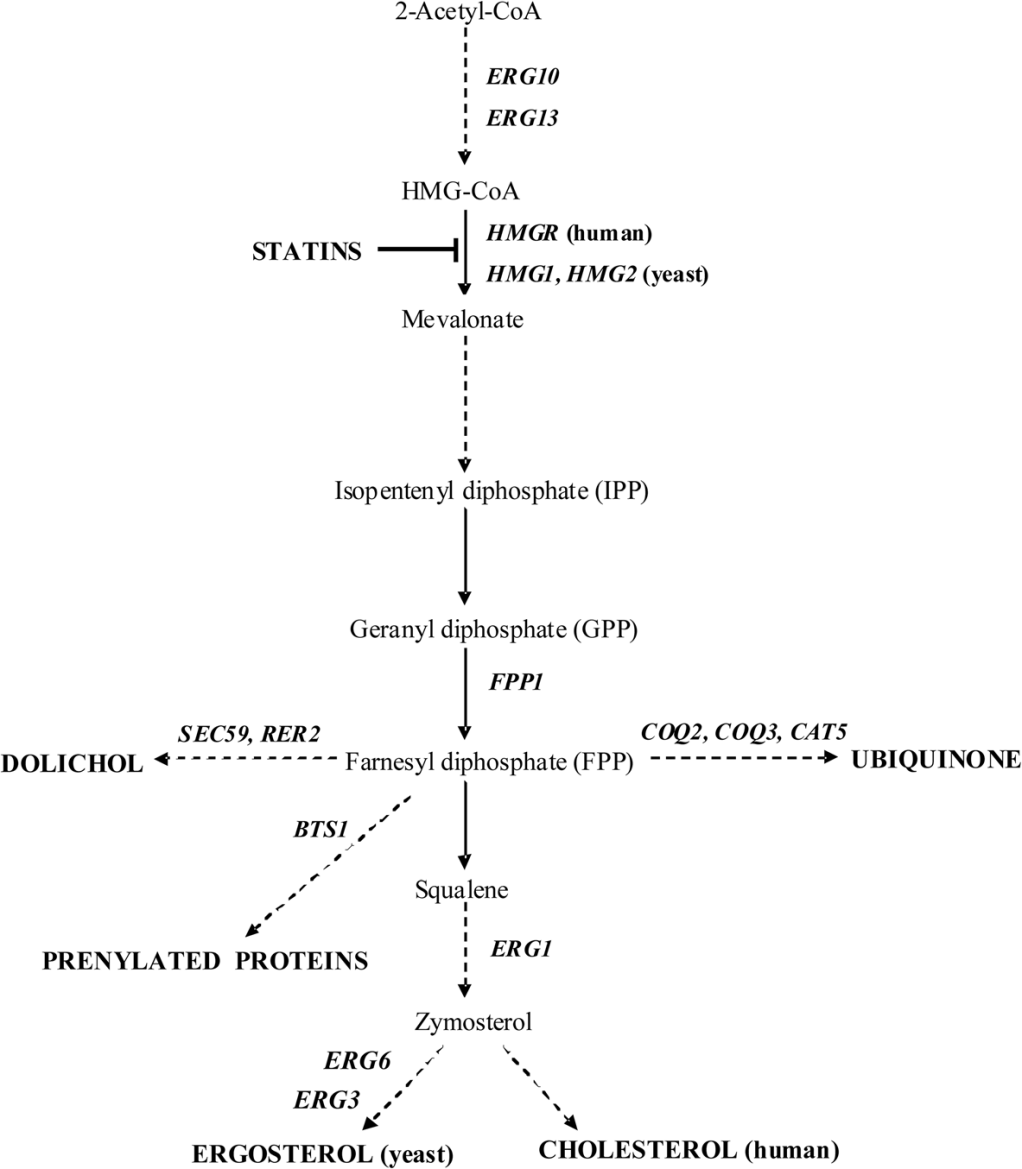
**The mevalonate pathway.** The enzymes selected for gene expression analysis are shown at the corresponding steps of the pathway. The dashed lines indicate multicomponent pathways. *ERG10* – acetyl-CoA acetyltransferase, *ERG13 –* 3-hydroxy-3-methylglutaryl-CoA synthase, human (*HMGR*) and yeast (*HMG1*, *HMG2*) 3-hydroxy-3-methylglutaryl-Co A reductase 1 and 2, *FPP1 –* farnesyl pyrophosphate synthase, *ERG1 –* squalene monooxygenase, *ERG6 –* delta(24)-sterol C-methyltransferase, *ERG3 –* C-5 sterol desaturase, *COQ3* – 3,4-dihydroxy-5-hexaprenylbenzoatemethyltransferase, *COQ2* – *para*-hydroxybenzoate-polyprenyl transferase, *CAT5* – ubiquinone biosynthesis monooxygenase, *BTS1* – geranylgeranyl diphosphate synthase, *RER2* – *cis*-prenyltransferase, *SEC59* – dolichol kinase.

The mevalonic acid pathway (MVA, Figure 1) leads to the synthesis of sterol isoprenoids, with the final product cholesterol (ergosterol in yeast), and nonsterol isoprenoids, such as dolichols, the side chain of ubiquinone, farnesyl diphosphate (FPP) and geranylgeranyl diphosphate (GGPP). Statins are well-known inhibitors of 3-hydroxy-3-methylglutaryl-coenzyme A reductase (HMGR). This group of drugs is widely used in the treatment of hyperlipidemia and cardiovascular diseases [1-3]. However, the effects of statins extend beyond their cholesterol-lowering action. Inhibition of HMG-CoA reductase, the regulatory enzyme of the pathway, results in disturbances in practically all vital cellular processes, such as protein glycosylation and prenylation, cell signaling, functioning of the respiratory chain and integrity of cellular membranes [4]. The impairment of these processes may contribute to the pleiotropic side-effects of statins [5-7].

The yeast *Saccharomyces cerevisiae* is a valuable model for studying metabolic pathways and cellular mechanisms of human diseases as it is genetically tractable and shares many similarities with human cells [8]. As a eukaryote, *S. cerevisiae* has many of the advantages of higher eukaryotic expression systems such as protein processing, protein folding and posttranslational modifications, at the same time being as easy to manipulate as are bacteria. The use of yeast as a model of fundamental cellular processes and metabolic pathways of the human has improved the understanding and facilitated the molecular analysis of many disease-related genes. Comparative genomics studies have shown that 40% of yeast proteins have at least one human homolog and 30% of genes involved in human disease pathology have an ortholog in yeast [9]. Also, many regulatory pathways are conserved between yeast and humans. For example, the use of recombinant yeast to screen for new inhibitors of human acetyl-CoA carboxylase has led to the discovery of potential drugs to treat obesity [10]. The mevalonate pathways in yeast and humans are highly similar. All the steps from HMG-CoA formation to zymosterol synthesis are biochemically the same. The high degree of conservation of the mevalonate pathway – from unicellular organisms to human cells – justifies the use of *S. cerevisiae* to study the general principles of this pathway and its response to drugs [9].

Here, we investigated the effects of four statins widely used in the clinical practice – simvastatin, atorvastatin, fluvastatin and rosuvastatin – on the growth rate and metabolism of yeast cells. Three yeast strains were constructed to express human HMGR or either of the yeast isoenzymes. We examined the levels of ergosterol – the yeast cholesterol analog – and its precursors after statin treatment. We also investigated the statin-induced changes in the expression of genes from the nonsterol branches of the MVA pathway such as those involved in ubiquinone and dolichol biosynthesis and in protein prenylation. The impact of individual statins on the expression of genes encoding enzymes of the sterol biosynthesis pathway was analysed by real-time PCR. The effects of statins on the levels of selected proteins from those pathways were investigated by Western blotting. Finally, we evaluated the effects of the individual statins on the mevalonate and associated pathways.

## Results

### The effects of statins on yeast growth

In this study we used a yeast expression system described earlier [11]. Here we applied the following strains:

• H – yeast strain with double *hmg1*Δ *hmg2*Δ deletion (i.e., completely devoid of native HMGR activity) transformed with a plasmid carrying the human *HMGR* gene.

• Y1 – double *hmg1*Δ *hmg2*Δ deletion yeast strain transformed with a plasmid carrying yeast *HMG1* gene.

• Y2 – double *hmg1*Δ *hmg2*Δ deletion yeast strain transformed with a plasmid carrying yeast *HMG2* gene.

Since the HMG-CoA reductase activity is essential for yeast growth, we could investigate the relative sensitivity of the human HMGR to the various statins by comparing the growth kinetics of appropriate recombinant yeast strains in the presence of different concentrations of the statins. Based on OD_600_ readings growth curves were plotted for each culture. For further research we chose the concentration of 100 μM for all statins, which was the highest non-toxic dose for yeast cells.

**Figure 2:**
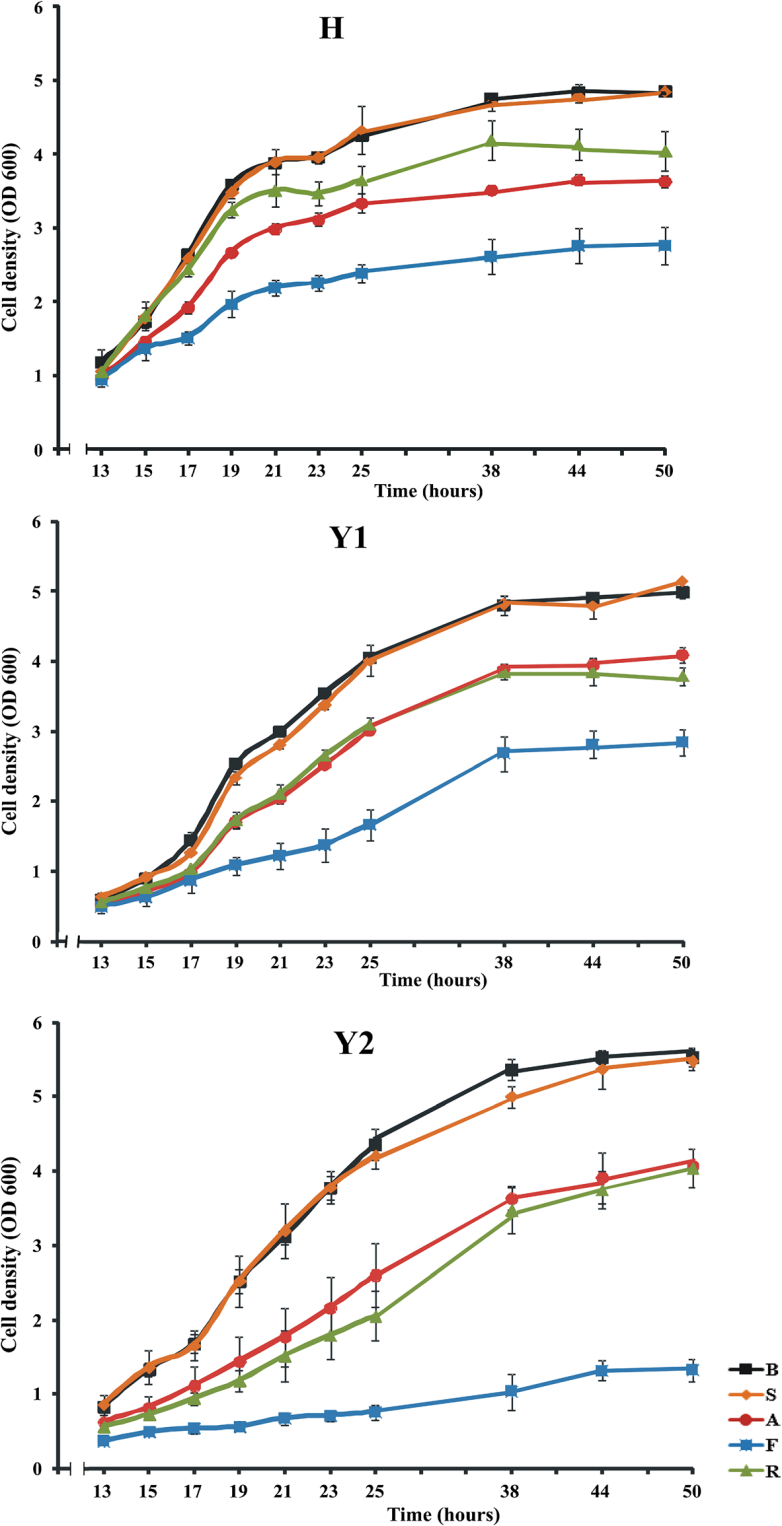
**Statins inhibit growth of yeast strains.** Growth kinetics of yeast strains cultured in liquid minimal media supplemented with either statins or buffer. OD_600_ of each culture was measured at intervals. Data represent mean ± SD of triplicate experiments. When the error bars are absent; SD falls within the size of symbols. B – buffer, S – simvastatin, A – atorvastatin, F – fluvastatin, R – rosuvastatin.

As shown in Figure 2, statins exerted different effects on the yeast strains tested. The strongest inhibitory effect on yeast growth was observed in cultures supplemented with fluvastatin. Atorvastatin and rosuvastatin caused milder yeast growth inhibition, whereas simvastatin only slightly affected yeast growth.

### Quantification of mRNA for genes encoding selected enzymes from the sterol and nonsterol branches of the isoprenoid biosynthesis pathways

The effects of statins on the level of expression of genes encoding enzymes involved in isoprenoid biosynthesis were investigated for selected enzymes whose homologs can be found both in yeast and human cells. These included:

• genes from the mevalonate pathway: acetyl-CoA acetyltransferase (*ERG10*), hydroxymethylglutaryl-CoA synthase (*ERG13*), human (*HMGR*) and yeast (*HMG1*, *HMG2*) 3-hydroxy-3-methylglutaryl-coenzyme A reductase, farnesyl diphosphate synthase (*ERG20, FPP1*).

• sterol-specific genes encoding enzymes involved in sterol biosynthesis: squalene monooxygenase (*ERG1*), C-5 sterol desaturase (*ERG3*) and, additionally, the gene coding for delta(24)-sterol C-methyltransferase (*ERG6*), an enzyme absent in humans (the encoded enzyme converts zymosterol to fecosterol in the pathway leading to ergosterol, the principal sterol in fungi).

• nonsterol-specific genes encoding enzymes involved in ubiquinone biosynthesis: *para*-hydroxybenzoate-polyprenyl transferase (*COQ2*)*,* 3,4-dihydroxy-5-hexaprenylbenzoate-*O-*methyltransferase (*COQ3*) and ubiquinone biosynthesis monooxygenase (*CAT5*); dolichol synthesis: *cis*-prenyltransferase (*RER2*) and dolichol kinase (*SEC59*); and protein prenylation: geranylgeranyl diphosphate synthase (*BTS1*).

**Figure 3:**
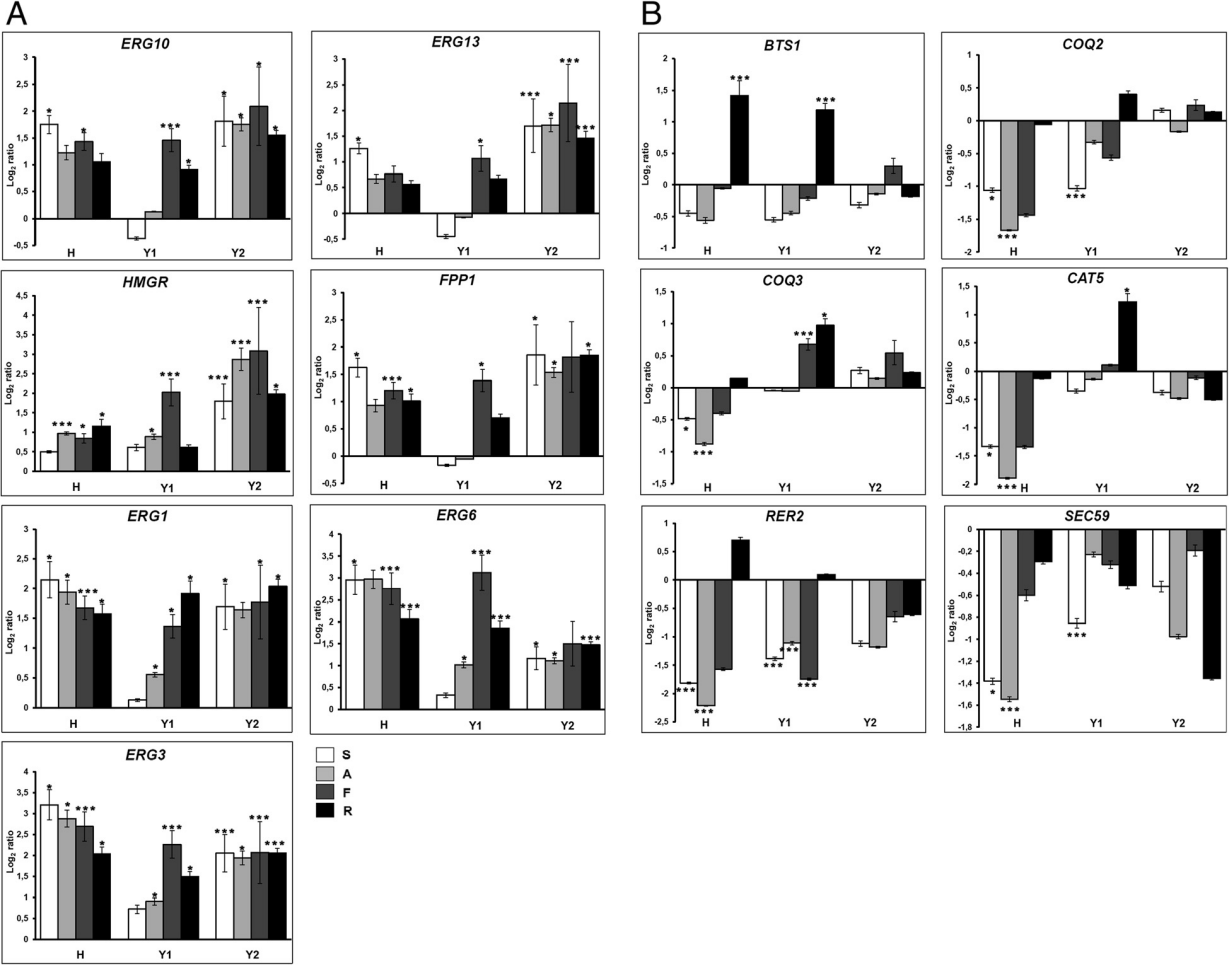
**Statins induce expression of genes encoding enzymes of sterol and nonsterol biosynthesis pathways.** mRNA levels in statin-treated cells relative to control (buffer-treated cells) are shown using log_2_ scale. Results are mean ± SEM obtained by qRT-PCR. *p < 0.05, ***p < 0.001. Real-time PCR data, were normalize to *35S rRNA*, a housekeeping gene. S – simvastatin, A – atorvastatin, F – fluvastatin, R – rosuvastatin. **A)** Quantitative real-time RT-PCR analysis of selected genes encoding enzymes of sterol biosynthesis pathway after statin treatment compared to buffer-treated cells. *ERG10* – acetyl-CoA acetyltransferase, *ERG13 –* 3-hydroxy-3-methylglutaryl-CoA synthase, *HMGR –* human 3-hydroxy-3-methylglutaryl-Co A reductase, *HMG1* and *HMG2* – yeast 3-hydroxy-3-methylglutaryl-Co A reductase 1 and 2, *FPP1 –* farnesyl pyrophosphate synthase, *ERG1 –* squalene monooxygenase, *ERG6 –* delta(24)-sterol C-methyltransferase, *ERG3 –* C-5 sterol desaturase. **B)** Quantitative real-time RT-PCR analysis of genes from ubiquinone and dolichol synthesis and protein prenylation pathways. *CAT5***–** ubiquinone biosynthesis monooxygenase, *COQ3* – 3,4-dihydroxy-5-hexaprenylbenzoate-*O*-methyltransferase, *COQ2* – *para*-hydroxybenzoate-polyprenyl transferase, *BTS1* – geranylgeranyl diphosphate synthase, *RER2* – *cis*-prenyltransferase, *SEC59* – dolichol kinase.

In general, the statins induced expression of the genes from the mevalonate pathway and its sterol-specific branch, although the magnitude of the stimulation differed greatly between the genes, and for a given gene, between the four statins (Figure 3A). In the H strain carrying human *HMGR* the strongest effect was observed after simvastatin treatment, except for the human *HMGR* gene itself, whose expression was induced to the highest degree after rosuvastatin treatment.

In contrast, statins caused highly divergent changes in the expression of genes coding for the enzymes from the ubiquinone synthesis (*COQ2, COQ3, CAT5*), dolichol synthesis (*RER2, SEC59*) and protein prenylation (*BTS1*) pathways (Figure 3B). In strain H expression of all those genes was reduced by simvastatin, atorvastatin and fluvastatin. Interestingly, rosuvastatin caused a diverse effect: expression of three genes (*BTS1*, *COQ3* and *RER2*) was induced, and three other genes (*COQ2*, *CAT5* and *SEC59*) responded with a slight decrease of expression. In the Y1 and Y2 strains expression of those genes was generally reduced, except for the *COQ3* gene. Notably, in the Y1 strain rosuvastatin induced the expression of all the tested genes. A striking conclusion from this part of the study is that the effects of rosuvastatin on the expression of genes from the nonsterol isoprenoid pathways are clearly different from those of the other statins, regardless of the yeast strain investigated. In the strain carrying human *HMGR* the action of rosuvastatin is more ‘positive’: it is either the strongest, or the only inducer of expression, or the weakest repressor.

### The level of selected proteins after statin treatment

**Figure 4:**
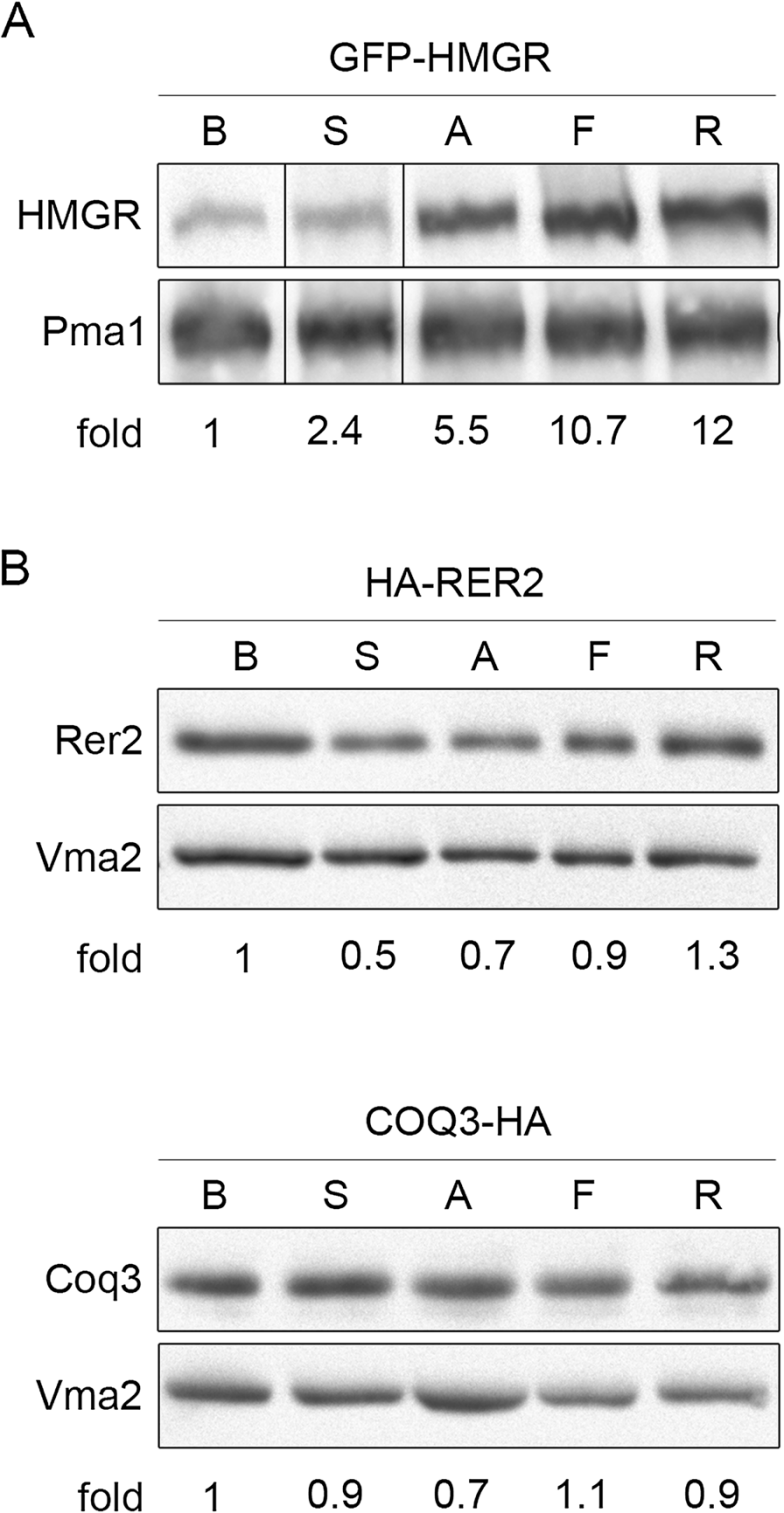
**Statins affect levels of human HMGR and yeast Rer2 and Coq3 proteins.** Strains expressing GFP-HMGR, HA-Rer2 or Coq3-HA proteins were grown for 24 hours in the presence of buffer (B) or one of the statins: atorvastatin (A), fluvastatin (F), rosuvastatin (R) or simvastatin (S). Protein extracts were prepared and analyzed by Western blotting. The level of plasma membrane ATPase Pma1p is shown as a loading control. **A)** Steady state level of human HMGR is higher after statin treatment with the highest increase after rosuvastatin treatment. Western blot was analyzed with anti-GFP or, for loading control, anti-Pma1 antibody. Ratio of respective signals is shown below. **B)** Levels of yeast Rer2 and Coq3 proteins are marginally affected by statin treatment. Western blots were analyzed with anti-HA or, for loading control, anti-Vma2 antibody.

In parallel to the transcript analysis Western blotting was applied to follow the cellular levels of corresponding proteins. As shown in Figure 4, the level of human HMGR protein was higher after statin treatment compared to the control. The magnitude of that effect depended on the type of statin used: the lowest accumulation was observed after simvastatin treatment (about 2.4-fold) and the highest one – in the presence of rosuvastatin (about 12-fold). In contrast to the high increase of the human HMGR protein level after statin treatment, the levels of the nonsterol pathway proteins, Rer2 and Coq3, were only slightly changed in response to the statins. In general, the response of those two enzymes at the protein level correlated well with the effects of the statins on their mRNA levels.

### Efficiency of individual statins in decreasing the level of sterols

To investigate the effects of various statins on yeast cell sterol levels, the H strain was treated with the four statins or buffer, sterols were isolated and submitted to GC/MS analysis. The levels of ergosterol and its precursors squalene, lanosterol, zymosterol and fecosterol, as well as the total amount of identified sterols, were calculated. In statin-treated cells the total sterol pool and the levels of each sterol precursor identified were lower than in control cells (Table 1). The most prominent decrease was noted for squalene, the content of which was between 24% (after atorvastatin) and 7% (after fluvastatin) of its content in control cells. Of the sterols measured, the level of ergosterol was the least diminished (to between 13% of the control level after simvastatin and 54% after rosuvastatin). The four statins differed substantially in their overall inhibitory effects on sterol synthesis. Simvastatin exerted the strongest inhibitory effect (total sterol pool reduced to 13% of the control level) and rosuvastatin the mildest (total sterols lowered to 39% of the control).

**Table 1:** Levels of sterols and squalene after statin treatment in H strain

	**Simvastatin [%]**	**Atorvastatin [%]**	**Fluvastatin [%]**	**Rosuvastatin [%]**
Squalene	14	24	7	9
Lanosterol	10	23	12	12
Zymosterol	7	50	19	41
Fecosterol	7	22	15	16
Ergosterol	13	37	39	54
Total	13	33	27	39

Data expressed as percentage of the amounts found in control (buffer–treated) cells.

## Discussion

The use of yeast as a model organism for studying cell biology and a host for the expression of heterologous proteins is becoming increasingly popular in recent years [12,13]. In this study a yeast expression system was applied to investigate the effects of four statins widely used in the clinical practice – simvastatin, atorvastatin, fluvastatin and rosuvastatin – on a number of cellular processes. First, the effects of the statins on yeast growth were checked. The strongest inhibitory effect was exerted by fluvastatin, milder effects were noted for atorvastatin and rosuvastatin, whereas simvastatin did not significantly affect yeast growth. Those results suggest that at the dose used fluvastatin is the most toxic while simvastatin is the safest of the HMGR inhibitors tested.

As statins are mainly prescribed to hypercholesterolemic patients to normalize the levels of serum lipoproteins, we focused on expression of the genes related to sterol biosynthesis. A comparative analysis of expression of genes from the mevalonate pathway (*ERG10*, *ERG13*, *HMG1* and *HMG2*, *FPP1*) as well as sterol-specific genes encoding enzymes involved in sterol biosynthesis (*ERG1*, *ERG3* and, *ERG6*) revealed increased mRNA levels for most of those genes in response to statins. Statins also induced the expression of the HMGR-encoding genes (human *HMGR*, yeast *HMG1* and *HMG2*) in the respective strains. However, the magnitude of the response varied substantially between the four statins and also between the strains. In the H strain expressing human HMGR, simvastatin was the strongest inducer of all the above genes except for human HMGR itself, for which it was the weakest inducer of all the statins assayed. Our results indicate that the HMGR inhibitors affect the expression of practically all enzymes of the sterol branch of the mevalonate pathway and this effect depends on the type of statin administered but also, rather unexpectedly, on the type of HMGR expressed in the cell.

In general, in response to statin treatment cells develop an adaptive response compensating for the diminished level of sterols which include increased expression of mevalonate pathway genes. Similar results were observed in human skeletal muscle-like cells [14] and human hepatoma HepG2 cells [15]. In those studies statins increased the expression of numerous cholesterol synthesis-related genes. This is in accordance with the results of Gerber et al. [16], Hagemenas and Illingworth [17] and ours [18] proving that inhibition of HMGR activity by statin treatment stimulates an adaptive response in the cell in order to maintain sterol homeostasis, including up-regulation of genes involved in cholesterol biosynthesis, a compensatory increase in HMGR level and consequent partial reduction of the sterol lowering effect.

To investigate whether statins produce similar effects on the expression of genes involved in pathways branching off the main sterol biosynthesis pathway we chose six genes involved in ubiquinone synthesis (*COQ2*, *COQ3* and *CAT5*), dolichol synthesis (*RER2*, *SEC59*) or protein prenylation (*BTS1*). Surprisingly, the effects of statins on the expression of those genes were unlike those discussed above for the main pathway. Highly divergent changes in the expression of the genes tested were noted after statin treatment, with the majority showing reduced level of transcripts.

Our mRNA quantification results show that statins generally decrease expression of ubiquinone synthesis-related genes in the H strain (*CAT5*, *COQ2*, *COQ3*). The strongest such decrease was noted in yeast cells treated with atorvastatin. Experimental data showed that atorvastatin significantly decreases the coenzyme Q level in the blood of patient at risk for cardiovascular disease and stroke [19,20]. Ample studies have demonstrated that, as a consequence of HMG-CoA reductase inhibition, statins block the production of farnesyl diphosphate (FPP), serving as substrate for, among others, the synthesis of the side chain of ubiquinone. The direct effect through FPP depletion may not be the only mechanism whereby statins lower CoQ synthesis.

Next, we checked the effects of statins on the expression of genes encoding yeast *cis*-prenyltransferase Rer2p and dolichol kinase Sec59p*.* In our study statins generally reduced expression of *RER2* and *SEC59* genes, which suggests that the dolichol synthesis could be decreased in statin-treated cells. Polyprenols and dolichols (polyisoprenoid alcohols with saturated α-residue) are long-chain highly hydrophobic lipids broadly distributed in all tissues and cellular membranes of eukaryotic cells. Moreover, dolichol affects the structure and fluidity of the cellular membrane and probably also influence the activity of membrane-associated proteins [21] or protect components of biological membranes against oxidative stress [22]. Dolichol has also been shown to delay the G1 cell cycle arrest in human fibroblasts [23]. Furthermore, dolichol plays a role in the regulation of angiogenesis during tumor growth [24].

In the protein prenylation branch we observed a significantly increased geranylgeranyl diphosphate synthase (*BTS1*) mRNA level after rosuvastatin treatment. Protein prenylation is crucial for global cellular functions such as proliferation, differentiation, apoptosis and carcinogenesis. It requires the 15-carbon isoprenoid farnesyl diphosphate (FPP) or the 20-carbon geranylgeranyl diphosphate (GGPP), both of which are intermediates of the MVA pathway. Prenylated Rho and Rac proteins are potent negative regulators of endothelial nitric oxide synthase (eNOS). By inhibition of geranylgeranylation statins were found to inactivate the Rho and Rac proteins, causing eNOS up-regulation and subsequent NO release [25]. Moreover, inhibition of Rac1 protein in the course of statin therapy accounted for a decreased development of cardiac hypertrophy [26].

Western blotting analysis showed clearly that statin treatment increases the steady state level of human HMGR protein in the H strain of yeast. The strongest increase was observed in rosuvastatin-treated cells, which is in accordance with the results of the gene expression analysis which showed the highest induction of human *HMGR* gene expression after rosuvastatin treatment. In contrast to the substantial increase of the human HMGR protein level after statin treatment, the levels of Rer2 and Coq3 proteins were only slightly changed in statin-treated cells. The response of Rer2 and Coq3 proteins is similar to the changes of the their transcripts level after statins treatment.

HMG-CoA reductase inhibitors are highly potent at lowering LDL cholesterol. Here, statins substantially lowered the level of ergosterol and (in most instances) much more strongly the levels of all its triterpene precursors (Table 1). This is in accordance with the results of Fowler et al. [27] showing a profound decrease of ergosterol level and an even deeper depletion of its precursors in lovastatin-treated yeast cells. Both these results indicate that following HMGR inhibition the yeast cell, whether it expresses the native enzyme [27] or the human one (this work), increases the rate of precursor conversion into ergosterol in an attempt to maintain the supply of this vital sterol. It remains to be shown whether the same is also true of human cells. If so, the inhibition of HMGR by statins would result in a deeper depletion of cholesterol precursors than of cholesterol itself. Such a disproportionately profound depletion could possibly account for some of the adverse effects of statin therapy [28].

In our study simvastatin caused the strongest reduction in the content of sterols in the strain carrying human HMG-CoA reductase. This observation correlates with the gene expression results which showed the strongest overexpression of genes encoding selected enzymes from the sterol-specific branch after simvastatin treatment in the H strain. On the other hand, rosuvastatin differed from the other statins in its effect on the mRNA level of nonsterol-specific genes. In the strain carrying human *HMGR* expression of genes from nonsterol isoprenoid biosynthesis branches was strongly reduced after simvastatin, atorvastatin and fluvastatin treatment, while rosuvastatin caused only a mild repression or, for some of those genes, even a substantial induction. Taken together, this proves that statins, although classified to the same therapeutic group, differ strikingly in terms of their potency of action on the cell metabolism.

It may be assumed that statins affect the cell on several metabolic levels. The presented data suggest that inhibition of HMGR activity by statins stimulates an adaptive response in the cell, including up-regulation of genes involved in sterol biosynthesis. Genes from the pathways branching off the sterol-synthesis route appear to be less susceptible to the up-regulation, possibly because the levels of substrates for their enzymes are lower after statin treatment. Moreover, the diverse effects observed for individual HMGR inhibitors could be of major importance in the clinical practice, as the statins are prescribed to patients of various health conditions and different settings of cardiovascular diseases.

## Conclusions

The presented yeast expression system is suitable for studying the effects of HMG-CoA reductase inhibitors on various cellular processes, such as sterol biosynthesis, gene expression and protein levels. We have shown that the statins differ in their potency of action on gene expression, protein levels and lipid content. They induce expression of genes from the main sterol biosynthesis pathway. Genes from the pathways branching off the main one appear to be less susceptible to upregulation. Statin treatment substantially reduces the overall level of cell sterols (depending on the statin, between 3-fold and almost 8-fold), with the final product ergosterol being less affected than its precursors.

## Methods

### Yeast strains and plasmids

All the yeast strains used in this study were *S. cerevisiae* strains in the BY4742 background. Haploid yeast strain H was derived from MB03-1D in which double deletion of both genes encoding yeast HMG-CoA reductases, *hmg1*Δ and *hmg2*Δ, was complemented by expression of human *HMGR* gene introduced on the YEp351 plasmid [11,18]. Additionally, strains Y1 and Y2 were constructed in which the *hmg1*Δ *hmg2*Δ double deletion was complemented by yeast *HMG1* or *HMG2* genes, respectively, introduced on YEp351 plasmid.

The YEp351 plasmid derivative for expression of the human HMG-CoA reductase was made in the following way. The *Sac*I – *Sal*I DNA fragment from pUG36 containing human *HMGR* gene [11] fused with an N-terminal yeGFP (yeast-enhanced green fluorescent protein) tag, under the control of the yeast MET25 promoter, was inserted into the YEp351 yeast expression plasmid.

To construct plasmid pYH1 for the expression of yeast *HMG1* gene, the *HMG1* gene was amplified by PCR with the following primers: F-SpeI-HMG1 5′-CTAGACTAGTATGCCGCCGCTATTCAAGG-3′ and R-BamHI-HMG1 5′-CGCGGATCCTTAGGATTTAATGCAGGTGACG-3′ containing recognition sequences for *Spe*I and *Bam*HI restriction enzymes. The amplified DNA fragment was cloned into pJet1.2 (Fermentas). The resulting plasmid was digested with *Spe*I and *Bam*HI, and the obtained fragment was inserted into the *Spe*I-*Bam*HI sites of the pUG36 yeast expression vector (Guldener and Hegemann, unpublished data) to obtain the pYH1 construct. pYH1 was digested with *Sac*I and *Sal*I, and the obtained fragment was inserted into the *Sac*I-*Sal*I sites of the YEp351 expression vector to give a construct encoding yeast HMG1 reductase fused with an N-terminal yeGFP (yeast-enhanced green fluorescent protein) tag, under the control of the yeast MET25 promoter.

To construct plasmid pYH2 for expression of yeast *HMG2* gene, the *HMG2* gene was amplified by PCR with the following primers: F-BamHI-HMG2 5′-CGGGATCCATGTCACTTCCCTTAAAAACGAT-3′ introducing a *Bam*HI site before the START codon and R-HMG2 5′-TTATAATAATGCTGAGGTTTTAC-3′. The amplified DNA fragment was cloned into pJet1.2 (Fermentas). The resulting plasmid served as a template for PCR amplification of the *HMG2* sequence with additional *Sma*I and *Sal*I flanking sequences, for which primers F-SmaI-BamHI-HMG2 5′-TCCCCCGGGCGGGATCCATGTCAC-3′ and R-SalI-HMG2 5′-ACGCGTCGACTTATAATAATGCTGAGGTT-3′ were used. The amplified DNA fragment was cloned into pJet 1.2 (Fermentas). The resulting plasmid was digested with *Sma*I and *Sal*I, and the obtained fragment was inserted into the *Sma*I-*Sal*I sites of the pUG36 yeast expression vector to obtain the pYH2 construct. pYH2 was digested with *Sac*I and *Sal*I, and the obtained fragment was inserted into the *Sac*I-*Sal*I sites of the YEp351 expression vector to give a construct encoding yeast *HMG2* reductase gene fused with an N-terminal yeGFP tag, under the control of the yeast MET25 promoter.

In order to obtain the H COQ3-HA strain the COQ3 ORF was tagged at the 3′ end with a sequence encoding the 6HA epitope in the BY4742 strain. The 6HA epitope tag together with the hphNT1 cassette were PCR-amplified from pYM14 [29]. Primers for the tagging (fTagCoq3 5′-GTACCTCCCATATCAAGGGTGGGTTGAGCACGATTGTTCCGATGTCGGTAATTATTTTATGGCTATTCAGAGACTGAATCGTACGCTGCAGGTCGAC-3′ and rTagCoq3 5′-CTGTACGTGAAAAAGTGTATATATATATATTTATATAAGAAGATATTTACAGTCAGATACCTACTTTTCGTTTGATTTCAATCGATGAATTCGAGCTCG-3′) were designed as previously reported [29], except they both contained 80 bases of homology to the designated site of recombination. The PCR product was transformed to the BY4742 strain. The correctness of the 6HA epitope tagging was then confirmed by colony PCR and Western blot analysis of hygromycin resistant transformants. Selected transformant was crossed with the H strain and the obtained diploids were sporulated and dissected on complete medium. For further analysis spore clones that were hygromycin and geneticin resistant, and contained appropriate auxotrophy markers were selected. They were confirmed by colony PCR and Western blot analysis. The obtained H COQ3-HA strain (Mat **a***his3Δ1 leu2Δ0 lys2Δ0 ura3Δ0 hmg1Δ::kanMX4:HIS3 hmg2Δ::kanMX4 [pMET25-yeGFP-hHMGR/LEU]*) which expressed functional, HA-tagged Coq3 protein.

The pR7-HA plasmid for expression of HA-Rer2p [30] was kindly provided by Prof. Akihiko Nakano from the RIKEN Institute (Japan).

### Chemicals and media

Solutions of various statins were prepared as follows: the active substances were extracted from medicine tablets using 0.5% dodecyl sodium sulfate in 0.01 M sodium phosphate pH 7.0 buffer according to The United States Pharmacopoeia USP26. Simvastatin, atorvastatin, fluvastatin and rosuvastatin were extracted as above from Zocor® (Merck & Co., Inc., Whitehouse Station, NJ, USA), Atorvox® (Pliva Krakow, Zakłady Farmaceutyczne S.A), Lescol® (Novartis Pharma GmbH) and Crestor® (AstraZeneca AB, Sweden) tablets, respectively. Stock solutions at 10 mg/ml were stored at −20°C. The media and the genetic and microbiological techniques were essentially as in [31].

### Selection of statin dose for yeast treatment

In order to choose the proper dose of statins for yeast experiments *S. cerevisiae* cultures in liquid minimal media supplemented with increasing concentration of statins, i.e. 3.125 μM, 6.25 μM, 25 μM, 100 μM and 200 μM were cultivated with shaking at 30°C. The optical density of the cultures was measured at two-hour intervals. Based on OD_600_ results growth curves of each culture were plotted.

### Yeast growth and assay conditions

For testing growth in liquid cultures, inocula of *S. cerevisiae* were added to liquid minimal media supplemented with one of the statins or buffer as a negative control. Cultures were grown with shaking at 30°C and OD_600_ was measured after 13 h and then every two hours. Each culture was assayed in triplicate and the results were averaged.

### RNA isolation and reverse transcription

Inocula of *S. cerevisiae* cells expressing HMGR genes were added to liquid minimal media supplemented with one of the statins or buffer. The cultures were grown with shaking at 30°C and collected after 24 h. Total RNA was isolated from yeast cells using the MagNA Pure Compact RNA Isolation Kit (Roche, Germany). Reverse transcription (RT) was performed in duplicate using the QuantiTect Reverse Transcription Kit (QIAGEN, Germany), according to the manufacturer’s recommendations.

### mRNA quantification

Quantification of mRNA for the genes encoding enzymes of the mevalonate and sterol biosynthesis pathway: *ERG10*, *ERG13*, *HMGR, FPP1, ERG1, ERG6* and *ERG3*, and of the nonsterol isoprenoid pathways: *BTS1, COQ2, COQ3, CAT5, RER2* and *SEC59* was carried out using Real-time Polymerase Chain Reaction (PCR). qPCR amplification was performed using a LightCycler 1.5 and LightCycler FastStart DNA Master SYBR Green I (Roche Diagnostics GmbH, Germany), according to the manufacturer’s instructions. The Pfaffl model [32] was used to determine the relative expression ratio of a target gene between tested and control samples. This method is based on the mean CP deviation of the control and sample groups, normalized to a reference transcript and with real-time PCR efficiency correction. The relative expression software tool (REST-MCS^©^ – version 2) [33] was used for calculations. Data normalization was carried out in relation to the transcript of the housekeeping *35S rRNA* gene. The sequences of all primers and the qPCR parameters are detailed in Table 2.

**Table 2:** Primer sequences and qRT-PCR conditions

**Gene**	**Primer sequence**	**Annealing (°C)**	**Product size (bp)**
***ERG10***	F: 5′-GTCTGTGCATCCGCTATGAA-3′	61	124
	R: 5′-CTGCTGGCATGTAGTATGGT-3′		
***ERG13***	F: 5′-TGGTAGAGACGCCATTGTAG-3′	61	153
	R: 5′-GCGTGTTCCATGTAAGAAGC-3′		
***HMGR***	F: 5′-ATGCTCACAGTCGCTGGATA-3′	61	199
	R: 5′-ACAGCCAGAAGGAGAGCTAA-3′		
***HMG1***	F: 5′-CCGTATCCATGCCATCCATC-3′	61	158
	R: 5′-GACGGCACAGGCAACTATTC-3′		
***HMG2***	F: 5′-CGCCATGCTTGATCTTCTCG-3′	61	121
	R: 5′-GGAGCACAGAGACAGTTCAC-3′		
***FPP1***	F: 5′-TACAACACTCCAGGCGGTAA-3′	61	166
	R: 5′-CATCGGCGACCAAGAAGTAA-3′		
***ERG1***	F: 5′-GTGTTATCGGTGACGCTCCTA-3′	61	122
	R: 5′-TTCACGGTCGCTGAAGTCTA-3′		
***ERG6***	F: 5′-AAGACCTGGCGGACAATGAT-3′	61	197
	R: 5′-AGCAGCAGTAACTTCCTTGG-3′		
***ERG3***	F: 5′-TCACGGCTAGTCTCAGCTAC-3′	61	134
	R: 5′-AACGGTCAACATCGACATCC-3′		
***BTS1***	F: 5′-TAGGGGACAAGGCTTGGATA-3′	61	168
	R: 5′-ACCAACGAATGGCCGTGG TG-3′		
***COQ2***	F: 5′-GTGTCTCGGCTGCCTAAGAA-3′	61	198
	R: 5′-GCCAGCACCTCTCATTACCA-3′		
***COQ3***	F: 5′-AGTGAGCGTCTTGGATGTTG-3′	61	183
	R: 5′-TCCAGAGCCTTGCACTCATA-3′		
***CAT5***	F: 5′-TGGCTTGTACTGAAGCTGTC-3′	61	195
	R: 5′-CATGCTTGATAGCGGTGTCT-3′		
***RER2***	F: 5′-GTCGATACGGCTACCGTGTT-3′	61	133
	R: 5′-ACTTACAGGCCAGCTCTCCA-3′		
***SEC59***	F: 5′-AAGGTATGGCCGCATTCGTT-3′	61	176
	R: 5′-CTAGCACTCCACTCAGTGTA-3′		
***35S rRNA***	F: 5′-TCGACCCTTTGGAAGAGATG-3′	60	203
	R: 5′-CTCCGGAATCGAACCCTTAT-3′		

*F, R* forward and reverse primers, respectively.

### Western blot analysis

Protein extracts were prepared by alkaline lysis according to [34]. The extracts were additionally sonicated in a water bath for 5 minutes before SDS-PAGE. YeGFP-tagged human HMGR protein was detected with anti-GFP antibody (Roche) and secondary anti-mouse HRP-conjugated antibody (DAKO). 6HA-tagged yeast *cis*-prenyltransferase Rer2p and 3,4-dihydroxy-5-hexaprenylbenzoate-*O*-methyltransferase Coq3p proteins were detected with anti-HA antibody (BAbCo) and secondary antibody as above. To normalize protein levels between samples rabbit anti-Pma1 (a gift from Prof. Ramon Serrano) or mouse anti-Vma2 and secondary antibodies as above were used. The proteins were detected using an enhanced chemiluminescent substrate for detection of HRP (Thermo Scientific). The intensity of bands was calculated with ImageQuant 5.2 software.

### Lipid extraction

Yeast cells were harvested, washed with water, carefully decanted, weighed and frozen. Thawed yeast were suspended in a mixture of chloroform:methanol (1:1) and homogenized by extensive vortexing in the presence of a silica gel powder (0.063–0.20 mm), yeast:silica gel 2:1, w/w. The mixture was agitated overnight at room temperature. The homogenate was centrifuged (10 min; 2500 rpm) and obtained pellet was extracted three more times with chloroform:methanol (1:1). Pooled supernatants were dried with a stream of nitrogen and remaining lipids were subjected to alkaline hydrolysis by incubation in a mixture of 7.5% KOH in ethanol:water:toluene (85:15:100, by vol.) for two hours at 95°C. The progress of reaction was followed on TLC (Silica gel 60 F_254_ plates) with toluene:ethyl acetate (9:1) as a developing system. Lipids were visualized with iodine vapor. Lipophilic, non-saponifiable products were extracted with diethyl ether, extracts were washed with water, organic phase was evaporated to dryness in a stream of nitrogen and lipids were dissolved in hexane. Samples were stored at −20°C prior to further analysis.

### GC/MS analysis of sterols

Gas chromatography–mass spectrometry analysis of sterols was performed without derivatization on a 7890A gas chromatograph (Agilent Technologies) coupled with a 5975C single quadrupole mass spectrometer (Agilent Technologies) equipped with a triple-axis detector and an electron ionization ion source. An HP 5-MS column (30 m × 0.25 mm, 0.25 μm) was used. Oven temperature was maintained at 150°C for 5 min, and was then increased linearly to 300°C at a rate of 5°C/min. The temperature of the injector and the transfer line was 250°C. Helium was used as a carrier gas at a flow rate of 1 ml/min in the constant flow mode. Mass spectra were acquired in the scan mode (33 – 600 *m/z* range). Sterols were identified on the basis of their mass fragmentation patterns by comparing their spectra with those collected in the NIST Mass Spectral Program (NIST/EPA/NIH Mass Spectral Library Version 2.0f, build Oct 8 2008).

## Competing interests

The authors declare that they have no competing interests.

## Authors’ contributions

AL, AM coordinated and performed molecular studies; JK, IW, MWK, DT, MG, GS contributed to microbiological and protein analyses; JS, ES, MS, WD, AS were responsible for lipid analysis; DP, NO carried out data analyses; AL, AM, MG participated in design of study and drafted the manuscript; BB was responsible for the concept, supervision of the study and manuscript revision. All authors read and approved the final manuscript.
